# Novelty of Physiotherapy Protocols in a Classic Case of Extrapulmonary Tuberculosis in a 35-Year-Old Male Patient: A Case Report

**DOI:** 10.7759/cureus.57495

**Published:** 2024-04-03

**Authors:** Mayuri K Wanjari, Lajwanti Lalwani, Pooja R Tiwari

**Affiliations:** 1 Department of Cardiovascular and Respiratory Physiotherapy, Ravi Nair Physiotherapy College, Datta Meghe Institute of Higher Education and Research, Wardha, IND

**Keywords:** who-qol, six-minute walk test, physiotherapy protocols, physiotherapy, dyspnoea, extrapulmonary tuberculosis

## Abstract

Tuberculosis is the most frequent cause of death, specifically caused by a single infectious agent, *Mycobacterium tuberculosis*. There are two types of tuberculosis: pulmonary tuberculosis and extrapulmonary tuberculosis. Patients with extrapulmonary tuberculosis often have reduced lung function due to the disease's structural abnormalities, which also significantly impair their quality of life. The suggested standard of care for the treatment of extrapulmonary tuberculosis patients is pulmonary rehabilitation. A 35-year-old male patient who complained of shortness of breath, dry cough, and on-and-off fever diagnose with extrapulmonary tuberculosis was the subject of the case study. The patient had extrapulmonary tuberculosis with a history of pleural effusion, which was managed with proper medications. After increasing symptoms of the disease, the patient was referred for pulmonary rehabilitation. Physiotherapy protocol includes breathing exercises, relaxation techniques, and mobility exercises for the upper limb and lower limb. Effective physical rehabilitation was necessary to minimize complications and allow him to resume daily activities. Several outcome measures, like the dyspnea scale, visual analog scale, six-minute walk test, and World Health Organization-Quality of Life (WHO-QOL) questionnaire, were used to monitor the patient's progress during rehabilitation. The benefits of physiotherapy protocols emphasize the need for tailored approaches to addressing individual patient needs for comprehensive recovery as it significantly enhances clinical, physical, psychosocial, and overall quality of life, making it crucial for patients with extrapulmonary tuberculosis. The protocols are beneficial to improve exercise capacity, muscle force, symptoms such as dyspnea, cough, and health-related quality of life in these patients. In this study, the focus was more on breathing exercises such as segmental breathing exercises for lung expansion and increasing air entry in the lungs followed by improving functional capacity and strength.

## Introduction

Tuberculosis is the leading infectious agent-related cause of death and the ninth leading cause of death worldwide, ranking higher than HIV/AIDS. *Mycobacterium tuberculosis* infection affects 5%-15% of individuals, with higher rates in HIV-infected individuals and those with risk factors like smoking, alcoholism, diabetes, and malnourishment [1]. The most common type of tuberculosis, pulmonary tuberculosis, has substantial epidemiological significance due to its high contagiousness [2]. The percentage of patients with tuberculosis varies from nation to nation, which is influenced by variables such as location, social and ethnic traits, economic standing, and coexisting conditions [3]. According to the World Health Organization categorization criteria, a *Mycobacterium tuberculosis* infection that affects organs and tissues other than the pulmonary parenchymal is known as tuberculosis. It represents 20%-25% of all tuberculosis cases [4].

Extrapulmonary tuberculosis is the term for a single case of tuberculosis in a body part other than the lung. It involves spondylodiscitis, a rare but serious condition involving infection and inflammation of the intervertebral disc space and adjacent vertebrae, and is caused by common pathogens *Staphylococcus aureus* and *Escherichia coli*. This condition is characterized by severe neck and back pain, along with systemic symptoms like fever, malaise, general weakness, and weight loss. However, under the recommendations of the World Health Organization, patients diagnosed with pulmonary tuberculosis have been classified as having a locus [5]. Extrapulmonary tuberculosis is very common in underdeveloped nations, which suggests that individuals with chronic infections have high rates of both morbidity and mortality, resulting in poor life quality and health on a worldwide scale [6,7]. The World Health Organization deemed tuberculosis a dangerous, uncontrollable disease and a worldwide health emergency in 1933. It ranks among the top 10 causes of death worldwide, with an estimated 10.0 million new cases expected annually. It is divided into extrapulmonary and pulmonary tuberculosis. Pulmonary is different from extrapulmonary tuberculosis because a patient with pulmonary and extrapulmonary tuberculosis is classified as a case of pulmonary tuberculosis, for example, miliary tuberculosis is classified as pulmonary tuberculosis because there are lesions in the lungs. In developing nations, tuberculosis is still highly prevalent and deadly [8]. Extrapulmonary tuberculosis causes inadequate ventilation, incorrect gas exchange, and a decline in functional status. It also results in muscular atrophy and insufficient exercise capacity, which reduces exercise capacity, lowers daily activity, and reduces quality of life in terms of health. Spinal infection is a common symptom characterized by constant back pain, which is worse at night. Spinal infections may affect the vertebrae, discs, spinal canal, and paraspinal soft tissues [9].

Extrapulmonary tuberculosis has been treated with pulmonary rehabilitation. It is a comprehensive, non-pharmacological strategy that helps people with chronic pulmonary illnesses feel better both physically and psychologically. Rehabilitation programs may involve self-management, breathing exercises, teaching patients about their condition and its treatment, physical training that includes both aerobic and muscle-strengthening activities, and respiratory muscle training [10]. The physiotherapy management that reduces dyspnea provides breathing exercises, thoracic expansion, positioning, and inspiratory exercises with incentive spirometry. Exercises involving deep breathing are used to help individuals with extrapulmonary tuberculosis increase their lung volumes, oxygenation, and capacity. Another technique used to improve functional capacity is a six-minute walk test. It is frequently utilized to assess exercise capacity in lung disorders, including respiratory conditions. A six-minute walk test is used to assess a patient's functional ability if they have cardiac or respiratory problems [11]. The distance in a six-minute walk test on a level surface might be used here as an end measure for a rehabilitation program or a general one-time indicator of functional state. The six-minute walk test (6MWT) is a cost-effective, user-friendly, and repeatable test that accurately measures daily living activities, surpasses laboratory tests, and is well-received by patients [12].

## Case presentation

Patient information

**Figure 1:**
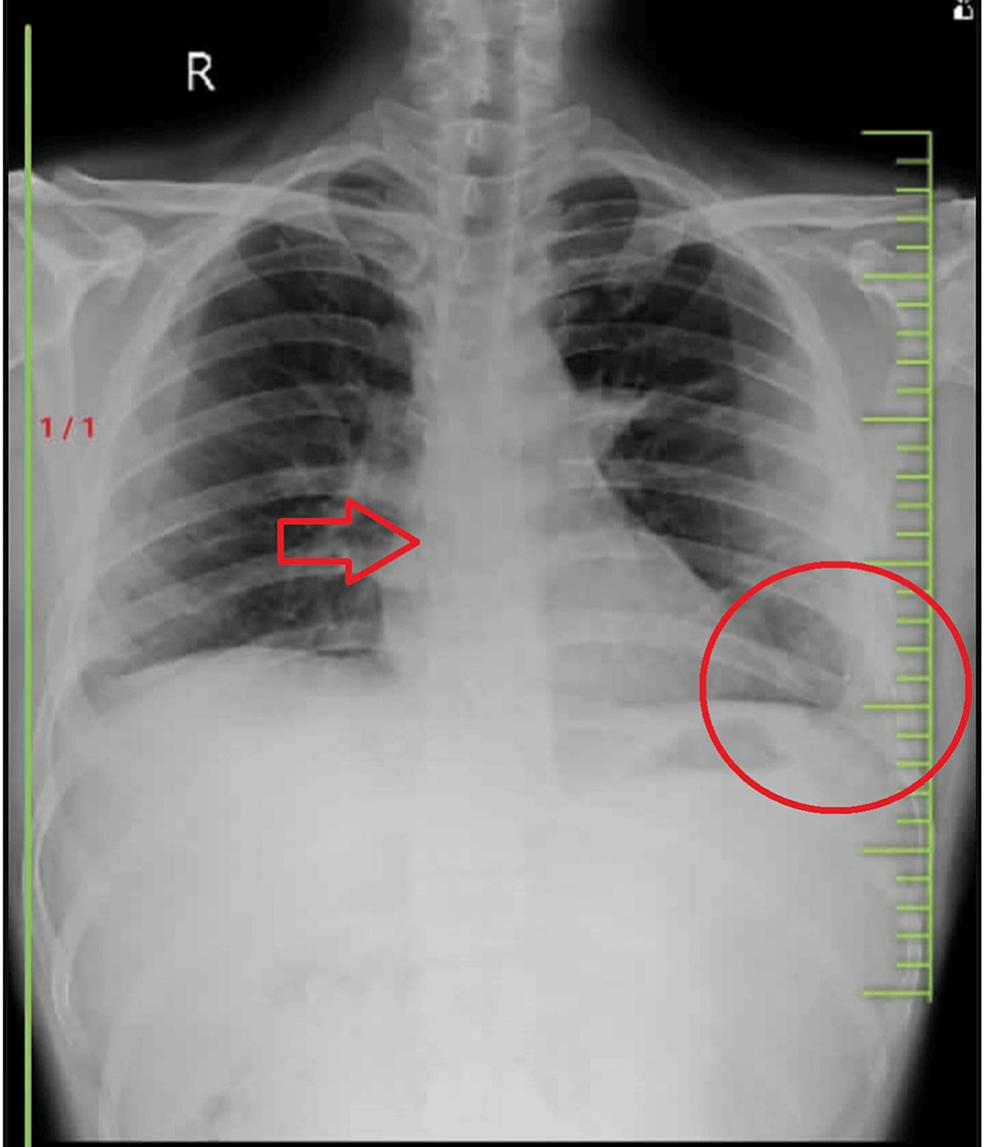
An anteroposterior view of the thorax X-ray shows an elevated angle, mild cardiomegaly, and mild cavitary lesions in the right middle and lower lung fields. The arrow shows the cardiomegaly, and the circle shows the cavitary lesion with bronchovesicular margins.

On January 20, 2024, a 35-year-old male patient from Mahagaon (Dist. Yavatmal) presented to the emergency care department with a complaint of on-and-off fever, dry cough, blurred vision, and shortness of breath that began slowly and persisted throughout the day, low back pain, bilateral lower limb pain, and easy fatigue for two weeks. The timeline is given in Table 1. The patient also stated that he had similar concerns four months prior, on September 19, 2023, when he went to a government hospital (Mahagaon), where he was advised to get an X-ray and a complete blood count test. Medication was given, but there was no relief, and the cough worsened. On December 5, 2023, the patient experienced the same symptoms and went to a private hospital (Yavatmal), where he was advised to get X-rays and sonography, which revealed tuberculosis with mild pleural effusion. After that, he recovered from the pleural effusion with conservative management. On January 20, the patient suffered the same symptoms and went to a multi-specialty hospital, where he completed the investigation, which included a chest radiography, as shown in Figure 1. Elevated angles, minor cardiomegaly, and mild cavitary lesions were detected in the right middle and lower lung areas. A full blood test revealed a low hemoglobin (8.3%) and a total WBC count of 3,700 per microliter; he was diagnosed with extrapulmonary tuberculosis and referred to cardiorespiratory physiotherapy for pulmonary rehabilitation.

**Table 1:** Timeline shows the date of events with description.

Sr. No	Events	Date of Events	Description of events
1.	Date of previous hospitalization	September 19, 2023	On-and-off fever, dry cough, and blurred vision.
2.	Date of visit to a private hospital	December 5, 2023	Increase in fever and cough. Diagnosed with tuberculosis.
3.	Date of pleural effusion	December 5, 2023	Recovered from pleural effusion.
4.	Date of emergency admission	January 20, 2024	Breathlessness, chest pain, and bilateral lower limb pain.
5.	Date of pulmonary rehabilitation	January 21, 2024	6-minute walk test, breathing exercises, and mobility exercises for the lower and upper limbs.

Diagnostic assessment

Thorax radiography is used for diagnosis.

Therapeutic intervention

Counseling and education for both patients and caregivers to lower the risk of integumentary complications of the respiratory system improve bed mobility and prevent prolonged immobility, promote airway clearance and alleviate dyspnea, increase lung volumes and capacities, prevent stiffness in the joints, and maintain their mobility and structural integrity, getting back to routine activities of daily living.

Physiotherapy protocol

**Figure 2:**
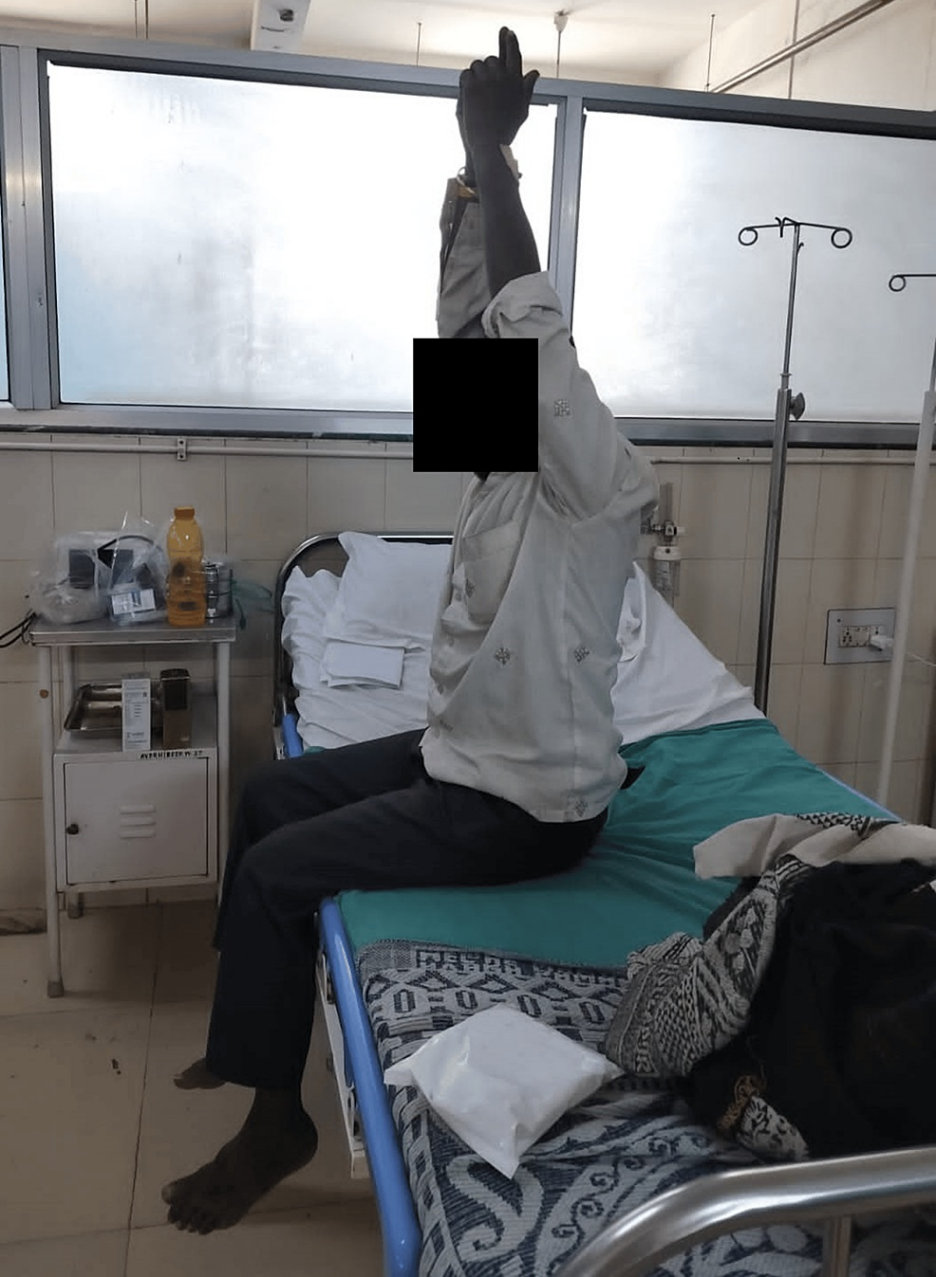
Bedside sitting and thoracic expansion exercise.

The basic purpose of pulmonary rehabilitation is to reduce symptoms. The main objectives of pulmonary rehabilitation are improved respiratory function, reduced back pain, increased range of motion, muscle strength, and enhanced quality of life. Table 2 shows the entire physiotherapy rehabilitation protocol with a two-week duration, which is a list of problems, goals, and management. Figure 2 shows the patient performing thoracic expansion and shows the patient performing dynamic quads in bedside sitting.

**Table 2:** Shows problem list, goal, and management. reps: repetitions.

Sr.no	Problem List	Goals	Management
1.	Lack of awareness about the disease	To give education about the disease and the role of physiotherapy patients and their families. Adherence to exercise is important.	The patient and his family were thoroughly educated about the disease and the significance of physiotherapy.
2.	Dyspnea	To reduce dyspnea	Dyspnea-relieving positions. Side-lying with a head elevation of 45˚ forward-leaning, sitting with a pillow on the bed.
		To improve ventilation	Controlled breathing: pursed lip breathing, diaphragmatic breathing, and deep breathing exercises with an additional breath hold of 5-7 seconds for improved breathing.
3.	Early or easy fatigue	To minimize early fatigue	Energy conservation techniques/pacing techniques. Pacing during work and taking breaks throughout the day when they feel fatigued.
4.	Reduced chest expansion	To improve chest expansion	Segmental breathing exercises followed by thoracic expansion exercises (5 reps 2 sets): so, the steps for thoracic expansion exercises are first, to take a larger-than-normal breath by holding the elbow, shoulder, and neck up. Then, we inhale by raising both upper limbs, letting go of the breath, and lowering the upper limbs simultaneously.
5.	Decreased strength	To increase the strength of the body and improve posture	Active range-of-motion exercises are employed for the lower and upper extremities when strength is reduced. Static sets of the hamstring, quad, and gluteal withholds lasting 5 seconds, then perform heel slides and ankle pumps. While the patient is acting, the therapist must oversee and monitor vital signs.
6.	Back pain and spasm	To reduce back pain and spasms	Hot fomentation for 15 mins. Followed by static sets. Back and glutes with 5 sec holds (10 reps × 1 set).
7.	Difficulty in doing activities of daily living.	Returning to regular activity of daily living	Walking at self-pace in a 10-meter hallway with assistance, spot marching, upper limb, and lower limb strengthening.

Outcome measure

The study's outcome measures include a modified Medical Research Council (mMRC) for dyspnea, a visual analog scale (VAS) for chest pain, a Hospital and anxiety depression scale for depression, and a six-minute walk test for functional capacity, listed in Table 3.

**Table 3:** Outcome measures for dyspnea, chest pain, depression, quality of life, and functional capacity. mMRC: modified Medical Research Council; WHO-QOL: World Health Organization-Quality of Life.

Outcome Measures	On the First day of Physiotherapy Reference	After 7 Days	After 14 Days of Discharge, in Follow-Up
Dyspnea (mMRC)	Grade III	Grade II	Grade I
Visual Analog Scale (for chest pain) On activity	6/10	4/10	2/10
Visual Analog Scale (for chest pain) on rest	3/10	2/10	0/10
Hospital and anxiety depression scale	9	7	4
WHO-QOL: Physical health	57/100	62/100	72/100
WHO-QOL: Emotional health	72/100	73/100	80/100
WHO-QOL: Social health	72/100	79/100	80/100
WHO-QOL: Environmental Health	70/100	78/100	80/100
6-min-walk test	180 m distance covered with a rest period of 3 min 20 sec	240 m distance covered with a rest period of 2 min	400 m distance covered with a rest period of 1 min

## Discussion

The study done by Jones et al. 2017 explains that extrapulmonary tuberculosis was recommended for cardio-respiratory physiotherapy to address additional problems such as dyspnea and upper and lower limb movement [13]. Extrapulmonary tuberculosis is characterized as a persistent condition that has a detrimental impact on a patient's social and physical well-being overall. The goal was to establish and evaluate a socially acceptable pulmonary rehabilitation (PR) program in Uganda for patients suffering from post-tuberculosis lung disease (p-TBLD). In a pre-post intervention research, patients with p-TBLD were assigned to a six-week, twice-weekly PR program. Recruitment, retention, the Clinical COPD Questionnaire (CCQ), exercise capacity testing, and biometrics were all used as outcome measures. In total, 34 participants began PR, and 29 (85%) finished full data collection. The average age of the 29 participants was 45 years, with 52% being female. The mean (95% confidence range) CCQ score at baseline was 1.8 (1.5, 2.0) after PR was 1.0 (0.8, 1.2), and six weeks later was 0.8 (0.7, 1.0). The Incremental Shuttle Walking Test (ISWT) measured 299 m (268.5, 329.4) at baseline, 377 (339.6, 413.8) after PR, and 374 (334.2, 413.5) six weeks later. Measures of chest pain improved; 13/29 (45%) participants reported chest discomfort at baseline, but only 7/29 (24%) after PR. Given that this was a development study, no formal statistical significance testing was performed [13]. Pulmonary rehabilitation is a comprehensive program based on data that has been shown to benefit individuals with extrapulmonary tuberculosis. Physiotherapy management consists of dyspnea-relieving positions, breathing exercises, thoracic expansion, inspiratory exercises using an incentive spirometer, and deep breathing exercises to enhance oxygenation and lung volumes [14]. The current study's findings corresponded to those of Sivaranjini et al. (2010), who explained that extrapulmonary tuberculosis patients had significantly decreased physical functional ability, particularly aerobic capacity, which has a considerable impact on cardiorespiratory endurance along with the main focus was to assess physical functional capacity (maximal oxygen consumption (VO_2_max)) in a group of older (50-65 years) patients with pulmonary tuberculosis and compare them to an age-matched healthy control group. A secondary goal was to create reference equations that may be used to forecast the six-minute walk test (6MWT) distance in older, healthy persons in India. The groups differed significantly in functional capacity and 6MWT distance (p < 0.001). Reference equations were created that used age, height, and weight to predict 6MWT distance in the healthy group. The complications of pulmonary tuberculosis have a significant influence on functional ability in older individuals in India [15]. The term "pulmonary rehabilitation" refers to the process of strengthening the functional capacity of the lungs using a six-minute walk test, which is used both as an evaluation tool and therapeutically in rehabilitation. Despite its limitations, 6MWD, together with symptom monitoring, functional class assessment, hemodynamic measures, and biological indicators, is an important tool for evaluating and treating pulmonary arterial hypertension (PAH) patients. However, recently completed trials involving patients on background treatments imply that clinical worsening accompanied by morbidity and death may be a more appropriate and meaningful primary end-point than a six-minute walk distance [16,17]. In our study, this assessment tool assisted us in determining our patient's optimal recovery. Because the patient stayed for such a short period, we provided aggressive physiotherapy protocols by increasing repetitions and sets and visiting him three times per day, with a primary focus on breathing exercises, functional ability, and strength training. The researchers discovered that patients with extrapulmonary tuberculosis performed better in the six-minute walk test than those with tuberculosis in earlier investigations [18]. The case study focuses on a patient's recovery from respiratory and cardiovascular issues using a two-week physical therapy regimen [19]. A well-structured physical therapy program, along with medicines, can speed up recovery and relieve symptoms in patients with extrapulmonary tuberculosis [20]. Specially designed management enhances a patient's quality of life by boosting mobility, stability, functional independence, and endurance [21].

## Conclusions

A well-planned, goal-oriented physiotherapy protocol, together with respiratory medications, contributes to the rapid recovery and reduction of symptoms for patients with extrapulmonary tuberculosis. The protocol, which included respiratory, musculoskeletal, and psychological components, was beneficial in improving patient recovery. The majority of therapeutic goals were achieved after two weeks of intensive physical therapy. This study highlights the benefits of physiotherapy protocol in ensuring patients' adequate and long-term recovery. Our goal began with psychological counseling and included breathing, relaxation, and mobility exercises for both upper and lower limbs. Effective physical therapy was required to reduce difficulties and allow him to resume normal activities. Several outcome measures, including the dyspnea scale, visual analog scale, six-minute walk test, and WHO-QOL questionnaire, were utilized to track the patient's improvement throughout rehabilitation. The benefits of physiotherapy protocols highlight the importance of tailored approaches to addressing individual patient needs for comprehensive recovery, as they significantly improve clinical, physical, psychosocial, and overall quality of life, making them essential for patients with extrapulmonary tuberculosis. These methods help to enhance exercise capacity, muscle force, symptoms like dyspnea and cough, and health-related quality of life in patients. In this study, the emphasis was on breathing exercises, such as segmental breathing exercises, to expand the lungs and increase air admission, followed by enhancing functional ability and strength.
